# Evaluation of FGL1 as a hepatokine marker in iron deficiency

**DOI:** 10.3389/fnut.2026.1767385

**Published:** 2026-03-06

**Authors:** Muhammad Saboor, Raghad Abdul Rahim, Shamsah Nabi Dad, Abeer Mohamed Yusuf, Mshael Mohammed Nasser, Hayat Mohsen Mansoor, Adnane Guella, Noura Alkhayyal

**Affiliations:** 1 Department of Medical Laboratory Sciences, College of Health Sciences, University of Sharjah, Sharjah, United Arab Emirates; 2 Research Institute for Medical and Health Sciences, University of Sharjah, Sharjah, United Arab Emirates; 3 Department of Clinical Sciences, College of Medicine, University of Sharjah, Sharjah, United Arab Emirates; 4 Department of Nephrology, University Hospital Sharjah, Sharjah, United Arab Emirates; 5 Medical Laboratory Department, University Hospital Sharjah, Sharjah, United Arab Emirates

**Keywords:** chronic disease, fibrinogen-like protein 1 (FGL1), hepatokine, iron deficiency anemia (IDA), iron metabolism

## Abstract

**Background:**

Fibrinogen-like protein 1 (FGL1) is a hepatokine that regulates hepcidin through antagonism of the bone morphogenetic protein (BMP) pathway. Although preclinical studies suggest a role for FGL1 in iron metabolism, its clinical behavior in human iron deficiency anemia (IDA) remains unclear. This study evaluates circulating FGL1 levels in IDA and examines its diagnostic performance and relationship with hematologic and biochemical markers.

**Methods:**

This cross-sectional study included 112 participants: healthy controls (*n* = 46), primary IDA (*n* = 46), and patients with IDA associated with chronic disease (IDA+CD) (*n* = 20). Hematologic indices, iron parameters, and serum FGL1 were measured. Group comparisons, correlation analysis, receiver operating characteristic (ROC) curves, and principal component analysis (PCA) were applied to assess diagnostic performance and multivariate biomarker structure.

**Results:**

Serum FGL1 concentrations were significantly higher in primary IDA (median 411.2 ng/ml) and IDA+CD (median 292.99 ng/ml) than in healthy controls (median 212.49 ng/ml; *p* < 0.001). Fibrinogen-like protein 1 did not differ significantly between IDA and IDA+CD (*p* = 0.106). In primary IDA, FGL1 showed weak correlations with iron markers, whereas in IDA+CD it demonstrated moderate associations with hemoglobinization indices, particularly MCH (*r* = 0.49). Receiver operating curve analysis showed excellent discrimination between healthy individuals and primary IDA (AUC 0.865) and good discrimination between healthy individuals and all disease groups combined (AUC 0.830). Fibrinogen-like protein 1 performed poorly in distinguishing primary IDA from IDA+CD (AUC 0.357). Principal component analysis showed that FGL1 clustered with classical markers of iron-restricted erythropoiesis along PC1, separating controls from both IDA groups.

**Conclusion:**

Fibrinogen-like protein 1 is markedly elevated in iron deficiency and aligns with the broader biochemical signature of iron-restricted erythropoiesis. Its strong ability to distinguish healthy individuals from those with iron deficiency suggests diagnostic potential, particularly when ferritin interpretation is limited.

## 1 Introduction

Iron deficiency anemia (IDA) remains the most common nutritional anemia worldwide, affecting an estimated 1.92 (1.89–1.95) billion individuals, with the highest burden observed in young children, women, and populations in low-resource settings (1). It contributes to impaired cognitive function, reduced physical capacity, and increased maternal-fetal morbidity (2, 3). Iron deficiency anemia develops when iron absorption, utilization, or intake fails to meet physiological requirements. Nutritional deficiency, chronic blood loss, malabsorption syndromes, menstrual disorders and pregnancy represent the major etiological factors (4). In contrast, chronic inflammatory and systemic disorders impair iron mobilization and reutilization, resulting in anemia of chronic disease/inflammation (ACD/AI) or a combined state of IDA with chronic disease (IDA+CD), where inflammatory cytokines inhibit erythropoiesis and disrupt iron recycling (5).

Iron homeostasis depends on coordinated regulation of intestinal absorption, macrophage recycling, and hepatic storage. Hepcidin serves as the central regulator of systemic iron metabolism (6). Its expression is primarily controlled by the Bone Morphogenetic Protein (BMP)–SMAD pathway in response to hepatic iron stores. Increased hepatocellular iron induces BMP6 synthesis, which binds to ALK2/3–BMPR2 receptors with hemojuvelin (HJV) functioning as an essential coreceptor (7). This interaction activates SMAD1/5/8, which complexes with SMAD4 and enters the nucleus to stimulate *HAMP* transcription. Elevated transferrin saturation further enhances signaling by stabilizing a functional HFE–transferrin receptor 2 (TfR2)–HJV complex on the hepatocyte surface (8). The resulting increased hepcidin promotes ferroportin degradation, limiting intestinal iron uptake and macrophage iron release (9).

In absolute iron deficiency (ID), low transferrin saturation disrupts the HFE–TfR2–HJV complex and attenuates BMP–SMAD signaling. Transmembrane Protease, Serine 6 [TMPRSS6 (also known as matriptase-2)] further suppresses this axis by cleaving membrane HJV. Loss-of-function TMPRSS6 variants produce inappropriately elevated hepcidin and iron-refractory iron deficiency anemia (IRIDA), underscoring its role as a key negative regulator (10). During erythropoietic stress, erythroblast-derived erythroferrone (ERFE) antagonizes BMP6 activity and lowers hepcidin to increase iron availability for erythropoiesis. Inflammation induces hepcidin synthesis through complementary cytokine-dependent pathways. Interleukin-6 (IL-6) activates JAK–STAT3 signaling, enabling phosphorylated STAT3 to bind the *HAMP* promoter and stimulate its transcription (11). Additionally, IL-1β enhances hepcidin synthesis via C/EBPδ induction (12). Furthermore, inflammation increases activin B, which signals through ALK2 and ActRIIA receptors to activate SMAD1/5/8 and further augment hepcidin transcription via BMP-responsive elements (13). These pathways collectively produce functional iron restriction, characterized by elevated hepcidin, ferroportin downregulation, and hypoferremia, the cardinal features of ACD/AI.

Beyond iron regulation, the liver acts as an endocrine organ that secretes hepatokines with systemic metabolic effects (14). Hepatokines such as fetuin-A and fibroblast growth factor 21 (FGF21) contribute to insulin resistance, dyslipidemia, and type 2 diabetes (15, 16). Fibrinogen-like protein 1 (FGL1) has emerged as a pleiotropic hepatokine involved in metabolic regulation, immune modulation, and carcinogenesis (17–20). Preclinical studies also identify FGL1 as a hypoxia-induced BMP antagonist that suppresses hepatic hepcidin transcription and promotes iron mobilization (21). This identifies FGL1 as a potential hepatokine regulator of iron metabolism, functionally parallel to the erythroblast-derived ERFE. However, its clinical relevance in human iron disorders, particularly across different etiological categories of IDA, remains unknown.

We hypothesize that FGL1 serves as a modulator in the pathophysiology of IDA. A comparative evaluation of FGL1 levels among healthy individuals, primary IDA, and IDA+CD may clarify its biological role. In addition, the associations between FGL1 and hematological indices, serum iron, and ferritin have not been fully defined. Establishing these relationships may determine whether FGL1 provides diagnostic or pathophysiological insight in states of absolute and functional ID.

The aims of this study were to: (i) compare circulating FGL1 concentrations among healthy controls, patients with primary IDA, and patients with IDA in the context of chronic disease (IDA+CD); (ii) investigate the associations between FGL1 and hematologic and iron-related indices as indicators of iron-restricted erythropoiesis; and (iii) assess the diagnostic performance of FGL1 and determine whether its expression differs between IDA alone and IDA occurring in chronic disease. This is the first clinical study to investigate FGL1 in human ID and seeks to translate preclinical findings into potential diagnostic or mechanistic relevance.

## 2 Material and methods

### 2.1 Study design and ethical approval

This comparative, cross-sectional study was conducted at University Hospital Sharjah, Sharjah, United Arab Emirates, between May and August 2025. The institutional Ethics and Research Committee approved the study protocol (UHS-HERC-176-16092043A). All procedures followed the principles of the Declaration of Helsinki.

### 2.2 Sample size calculation

The sample size was calculated *a priori* for the primary comparison between patients with IDA and healthy controls, with serum FGL1 as the primary endpoint. Using G^*^Power 3.1 software for a two-tailed independent *t*-test (α = 0.05, power = 80%), and a conservative large effect size (Cohen's *d* = 0.8) based on reported FGL1 differences in anemic patients (22), the calculation indicated a requirement of 52 participants per group. Recruitment feasibility and alignment with the reference study justified an enrolment of 46 participants in each group. A *post-hoc* power analysis confirmed that this sample size provides 78.5% power to detect an effect size of *d* = 0.8.

Patients with iron deficiency anemia in the context of chronic disease (IDA+CD) were identified from the same cohort, resulting in a smaller subgroup (*n* = 20). While the study was adequately powered for the primary comparison between IDA and healthy controls, the reduced size of the IDA+CD group limited the statistical power to detect moderate differences between IDA and IDA+CD. Accordingly, subgroup comparisons, correlation analyses, and diagnostic performance estimates involving the IDA+CD cohort were considered exploratory and were interpreted with caution.

### 2.3 Participant identification and cohort formation

Patients were identified retrospectively through a review of the institutional clinical laboratory database. Inclusion criteria for all anemia patients were: age ≥18 years, and laboratory-confirmed IDA, defined by hemoglobin below sex-specific reference intervals in conjunction with low serum iron and low serum ferritin. Iron deficiency anemia was defined using institutional laboratory reference limits. Anemia was defined as hemoglobin <13.5 g/dl in men and <12.5 g/dl in women. Iron deficiency was defined by reduced serum iron and ferritin concentrations below the laboratory reference ranges (serum iron: <11.6 μmol/L for men and <9.0 μmol/L for women; ferritin: <24 μg/L for both sexes). Exclusion criteria were active infection, malignancy, blood transfusion within the preceding three months, and laboratory profiles suggesting inflammation-mediated iron restriction rather than true ID. The latter included low serum iron in combination with elevated ferritin and/or raised C-reactive protein (CRP), to avoid bias from ferritin as an acute-phase reactant. Patients were stratified into two groups: primary IDA as patients without documented chronic inflammatory, metabolic, or systemic disease, and IDA+CD were individuals with comorbid chronic conditions, such as diabetes, chronic kidney disease, or osteoarthritis.

Eligible patients were contacted by telephone. The study objectives and procedures were explained, and verbal informed consent was obtained for the use of residual laboratory samples. 22 eligible individuals declined participation. The final cohort included 46 patients with primary IDA and 20 patients with IDA+CD.

### 2.4 Healthy control group

Forty-six healthy controls were recruited from hospital staff and community volunteers. Inclusion criteria required no history of anemia, chronic illness, or acute infection. Written informed consent was obtained from all controls. Venous blood samples were collected in EDTA tubes for hematology and in serum-separator tubes for biochemical analysis.

### 2.5 Sample handling and analysis

Serum FGL1 measurements were performed using residual samples obtained from the same blood draw that was used for routine hematological and iron profile testing. No additional or follow-up samples were collected for the purpose of this study. Sample identification, processing, and serum separation were completed within 4–6 h of collection. Because biomarker assessment was performed on the original diagnostic samples, no iron therapy, erythropoiesis-stimulating agents, or blood transfusion occurred between clinical testing and FGL1 measurement. Residual serum samples from patients were retrieved and stored at −80 °C until biomarker analysis. For healthy controls, serum was separated immediately after collection by centrifugation, aliquoted, and stored at −80 °C under the same conditions as patient samples. All samples were subjected to a single freeze-thaw cycle and were analyzed in batch under identical testing conditions to minimize analytical variability across study groups. Complete blood counts (CBC) were performed using a Mindray BC-6800 Plus (Shenzhen Mindray Bio-Medical Electronics Co., Ltd, China) hematology analyzer. Serum iron and ferritin measurements were obtained using the ARCHITECT c4000 (Abbott Laboratories, Illinois, USA) clinical chemistry analyzer. Serum FGL1 concentrations were quantified using a commercial enzyme-linked immunosorbent assay (ELISA) kit (USCN Wuhan, China), designed for quantitative detection of human FGL1. The assay has a detection range of 12.5–800 ng/ml with a minimum detectable concentration of <4.4 ng/ml. The assay demonstrates high analytical specificity with no significant cross-reactivity reported for related proteins. Manufacturer-reported precision indicates an intra-assay coefficient of variation (CV) <10% and an inter-assay CV <12%. Quantification was performed using a standard curve generated from serial calibrators supplied with the kit. Internal quality control procedures were performed in accordance with the manufacturer's recommendations. All assays were run in duplicate according to the manufacturer's instructions and absorbance was measured using a microplate reader.

### 2.6 Statistical analysis

Statistical analysis was performed using Python. The Shapiro–Wilk test assessed normality. Data are presented as mean ± standard deviation (SD) for normally distributed variables and median (interquartile range) for non-normally distributed variables. Categorical variables are presented as frequency and percentage. Comparisons among the three groups were conducted using one-way analysis of variance (ANOVA) with Tukey's *post-hoc* test for normally distributed data or the Kruskal–Wallis H test with Dunn's *post-hoc* correction for non-normal data. Correlations between FGL1 levels and hematological or iron parameters were evaluated using Pearson's or Spearman's correlation coefficients based on distribution. Diagnostic performance of FGL1 was evaluated using receiver operating characteristic (ROC) curve analysis, with calculation of area under the curve (AUC), sensitivity, and specificity. Principal component analysis (PCA) was applied to explore multidimensional patterns and to visualize clustering among the study groups based on hematological and biochemical parameters. The analysis included hemoglobin, RBC count, hematocrit, MCV, MCH, MCHC, RDW-CV, platelet count, WBC count, serum iron, ferritin, and FGL1. All variables were standardized (mean-centered and scaled to unit variance) prior to analysis to account for differences in measurement units. Components were retained based on variance explained and visual inspection of the scree plot. A *p*-value < 0.05 was considered statistically significant. All *p*-values are reported to three decimal places, and values < 0.001 are denoted as *p* < 0.001. Serum FGL1 demonstrated a non-normal distribution and was analyzed using non-parametric methods for group comparisons and Spearman's rank correlation for association analyses.

## 3 Results

### 3.1 Demographic characteristics of the study cohort

A total of 112 participants were enrolled: 46 patients with primary IDA, 20 patients with IDA+CD, and 46 healthy controls. Table 1 summarizes the demographic and clinical characteristics of the study population.

**Table 1:** Demographic and clinical characteristics of the study population.

**Characteristic**	**Healthy Controls (*n* = 46)**	**Primary IDA (*n* = 46)**	**IDA + CD (*n* = 20)**
Age (years)	–	–	–
Mean ± SD	47.8 ± 18.3	32.7 ± 16.7	60.7 ± 8.8
**Gender**, ***n*** **(%)**			
Female	29 (63.0%)	42 (91.3%)	16 (80.0%)
Male	17 (37.0%)	4 (8.7%)	4 (20.0%)
**Comorbid conditions**, ***n*** **(%)**^†^			
Diabetes mellitus	–	–	10 (50.0%)
Chronic kidney disease	–	–	6 (30.0%)
Osteoarthritis/joint disease^*^	–	–	6 (30.0%)
Gastrointestinal disease^∧^	–	–	4 (20.0%)
Hypothyroidism	–	–	3 (15.0%)
Ischemic heart disease (IHD)	–	–	1 (5.0%)
Liver disease	–	–	1 (5.0%)
Benign neoplasm	–	–	1 (5.0%)
Urticaria with pruritis	–	–	1 (5.0%)

^†^Conditions are not mutually exclusive; percentages represent the proportion of patients in IDA+CD (n = 20) with each condition. The sum exceeds 100% due to the presence of multiple comorbidities.

^*^Osteoarthritis/joint disease: includes patients with osteoarthritis (02), cervicalgia (02), spondylosis with radiculopathy (01) and intervertebral radiculopathy (01).

^∧^Gastrointestinal disease: includes patients with gastric ulcer, gastroenteritis, and gastrointestinal hemorrhage each.

Patients with the primary IDA were younger (mean age 32.7 ± 16.7 years) than both healthy controls (47.8 ± 18.3 years) and the IDA+CD group (60.7 ± 8.8 years). The primary IDA group showed a marked female predominance (90.6%). Gynecological causes constituted the most frequent etiology and included pregnancy (*n* = 6), anemia of puerperium (*n* = 3), irregular menstruation (*n* = 4), excessive menstrual bleeding (*n* = 3), and dysmenorrhea (*n* = 3). Gastrointestinal blood loss due to hemorrhoids (*n* = 4) was also observed. Idiopathic IDA represented the largest subgroup (*n* = 23), where no source of iron loss or cause of IDA was identified despite standard clinical assessment. The IDA+CD cohort was older and had a high burden of systemic disease. Diabetes mellitus (50.0%) and chronic kidney disease (30.0%) were the most frequent comorbidities. Female predominance remained evident (80.0%). Healthy controls demonstrated a more balanced sex distribution (63.0% female).

### 3.2 Comparative hematological and biochemical findings

Table 2 summarizes the hematological and biochemical features of the three cohorts. Both IDA groups demonstrated a classical hypochromic microcytic phenotype, with significantly reduced hemoglobin, serum iron, and ferritin levels compared with healthy controls (all *p* < 0.05).

**Table 2:** Descriptive statistics for key laboratory parameters across groups.

**Parameter**	**Normal (*n* = 46)**	**IDA (*n* = 46)**	**IDA+CD (*n* = 20)**	***p*-value**
Hb g/dL^†^	13.40 [12.90–14.20]	10.47 [9.70–11.20]	11.20 [9.95–11.35]	<0.001
RBC × 10^∧^12/L^†^	4.82 [4.43–5.10]	4.45 [4.09–4.75]	4.44 [3.90–4.90]	0.033
HCT %^†^	40.2 [37.4–43.6]	28.6 [24.7–32.3]	30.1 [26.5–33.8]	<0.001
MCV fL^†^	81.50 [79.10–88.10]	75.03 [70.10–79.80]	74.57 [68.90–80.60]	<0.001
MCH pg^†^	26.20 [24.70–29.60]	23.94 [21.50–26.20]	22.72 [20.30–25.40]	<0.001
MCHC g/dL^†^	32.20 [31.80–33.20]	31.80 [30.80–32.50]	31.07 [30.40–31.90]	0.001
RDW %^†^	13.70 [13.40–14.20]	15.91 [14.60–17.20]	16.50 [15.25–17.82]	<0.001
WBC × 10^∧^9/L^‡^	5.89 ± 1.40	6.69 ± 2.46	6.97 ± 1.23	0.049
Platelet × 10^∧^9/L^‡^	258.33 ± 51.26	283.53 ± 88.36	342.10 ± 101.90	0.001
Iron μmol/L^†^	18.55 [15.20–21.40]	5.89 [4.40–7.30]	7.60 [3.67–8.90]	<0.001
Ferritin μg/L^†^	42.00 [31.90–61.80]	4.90 [3.90–8.00]	8.51 [5.40–11.60]	<0.001
FGL1 ng/ml	212.49 [172.58–270.63]	411.2 [304.6–489.3]	292.99 [232.4–432.8]	<0.001

Data are presented as Mean ± Standard Deviation for normally distributed variables or Median [Interquartile Range] for non-normally distributed variables, as determined by the Shapiro-Wilk test. P-values are from Kruskal-Wallis (non-parametric) or one-way ANOVA (parametric) omnibus tests and are reported to three decimal places; values below 0.001 are indicated as <0.001.

Hb, hemoglobin; RBC, red blood cell count; HCT, hematocrit; MCV, mean corpuscular volume; MCH, mean corpuscular hemoglobin; MCHC, mean corpuscular hemoglobin concentration; RDW-CV, red cell distribution width-coefficient of variation; WBC, white blood cell count.

^†^Kruskal-Wallis test.

^‡^One-way ANOVA. Values are expressed in scientific notation as × 10^*n*^ per liter (e.g., RBC × 10^12^/L; WBC and Platelets × 10^9^/L).

**Figure 1:**
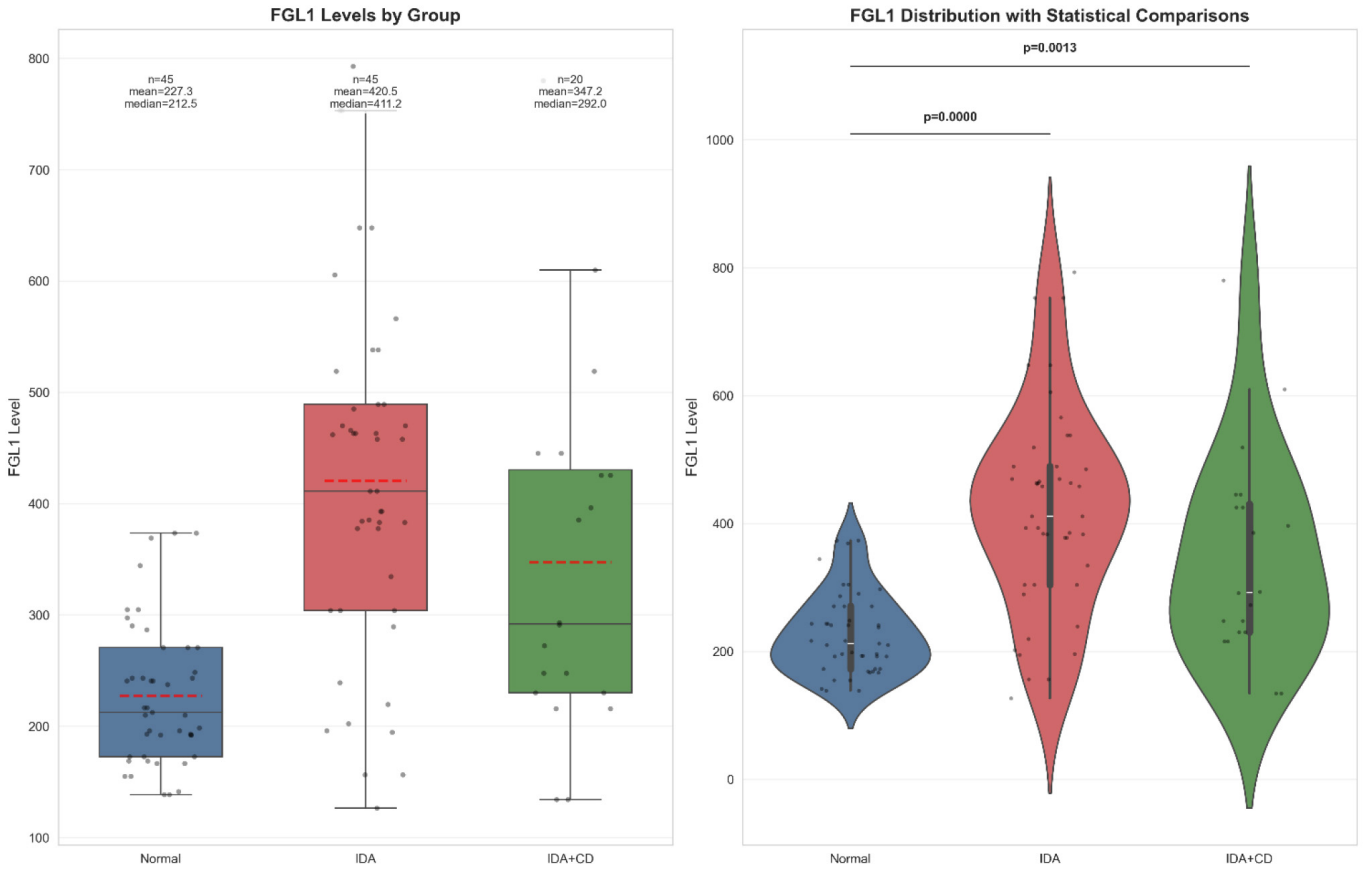
Serum FGL1 distribution across study groups. Distribution of serum FGL1 concentrations in healthy controls, patients with primary IDA, and patients with IDA+CD. Boxplots display median, interquartile range, range, individual data points, and group means (dashed line). Violin plots show the density distribution of FGL1 values with overlaid individual observations and pairwise statistical comparisons.

Serum FGL1 concentrations were significantly higher in both IDA groups than in healthy controls (Figure 1). Median FGL1 (ng/ml) levels were 212.49 in controls, 411.2 in primary IDA, and 292.99 in IDA+CD. The corresponding means were 227.3, 420.5, and 347.2, respectively.

Pairwise comparisons confirmed the significant elevation of FGL1 in both IDA groups vs. controls (*p* < 0.001 and *p* = 0.008, respectively) (Table 3). Although mean FGL1 was higher in primary IDA than IDA+CD, the difference did not reach significance (*p* = 0.106). Iron, ferritin, and hemoglobin did not differ significantly between the two IDA subgroups.

**Table 3:** Pairwise group comparisons of laboratory parameters.

**Parameter**	**Normal**		**IDA**		**IDA** + **CD**		***p*-value (N vs. IDA)**	***p*-value (N vs. IDA + CD)**	***p*-value (IDA vs. IDA + CD)**
–	**Mean** ±**SD**	**Median**	**Mean** ±**SD**	**Median**	**Mean** ±**SD**	**Median**			
Hb g/dL	13.69 ± 1.05	13.4	10.47 ± 1.14	10.7	10.38 ± 1.72	11.2	<0.001	<0.001	0.695
RBC × 10^∧^12/L	4.80 ± 0.52	4.82	4.45 ± 0.53	4.47	4.44 ± 1.01	4.53	0.033	0.941	1.000
HCT %	36.34 ± 3.76	35.5	33.05 ± 3.18	32.3	33.47 ± 5.14	35.5	<0.001	0.059	1.000
MCV fL	82.79 ± 6.46	81.5	75.03 ± 8.33	76.3	74.57 ± 10.06	73.05	<0.001	0.008	1.000
MCH pg	27.18 ± 2.96	26.2	23.94 ± 3.37	24.4	22.72 ± 3.65	21.9	<0.001	<0.001	0.530
MCHC g/dL	31.66 ± 3.01	32.2	31.32 ± 1.99	31, 8	31.07 ± 1.03	31	0.007	<0.001	0.308
RDW %	13.96 ± 0.92	13.7	15.91 ± 2.22	15.9	16.99 ± 2.51	16.5	<0.001	<0.001	0.944
WBC × 10^∧^9/L	5.89 ± 1.40	6.05	6.69 ± 2.46	6.26	6.97 ± 1.23	7.21	0.383	0.008	1.000
Platelet × 10^∧^9/L	258.33 ± 51.26	266	283.53 ± 88.36	272	342.10 ± 101.90	351	0.730	0.004	0.211
Iron μmol/L	18.55 ± 4.36	17.7	5.89 ± 2.13	5.6	6.42 ± 2.95	7.6	<0.001	<0.001	1.000
Ferritin μg/L	52.58 ± 29.50	42	6.31 ± 3.87	4.9	8.51 ± 4.63	7.25	<0.001	<0.001	0.073
FGL1 ng/mL	227.34 ± 63.09	212.49	420.55 ± 158.90	411.23	347.15 ± 163.08	292.99	<0.001	0.008	0.106

Data is shown as Mean ± SD.

Hb, hemoglobin; RBC, red blood cell count; HCT, hematocrit; MCV, mean corpuscular volume; MCH, mean corpuscular hemoglobin; MCHC, mean corpuscular hemoglobin concentration; RDW, red cell distribution width; WBC, white blood cell count.

p-values are from post-hoc pairwise tests following significant omnibus results (Mann-Whitney U with Bonferroni correction). P-values are reported to three decimal places; values below 0.001 are indicated as <0.001. Values are expressed in scientific notation as × 10^*n*^ per liter (e.g., RBC × 10^12^/L; WBC and Platelets × 10^9^/L).

The magnitude of these differences was quantified using effect size analysis and corresponding 95% confidence intervals are presented in Table 4. The increased levels of FGL1 in the primary IDA group compared to healthy controls represented a medium effect (*r* = 0.72). Notably, the difference in FGL1 levels between the primary IDA and IDA+CD groups also corresponded to a medium effect size (*d* = 0.62).

**Table 4:** Effect sizes for pairwise group comparisons of selected key parameters.

**Parameter**	**Comparison (Group1 vs. Group 2)**	**Effect Size**	**95% CI**	**Effect Size Name**	**Magnitude**
FGL1	Normal vs. IDA	0.72	0.55–0.85	Rank-biserial r	Medium
FGL1	Normal vs. IDA+CD	0.48	0.20–0.69	Rank-biserial r	Small
FGL1	IDA vs. IDA+CD	0.62	0.08–1.12	Cohen's d	Medium
Ferritin	Normal vs. IDA	−1.00	−1.00−0.90	Rank-biserial r	Large
Iron	Normal vs. IDA	3.69	2.95–4.43	Cohen's d	Large
Hemoglobin	Normal vs. IDA	−1.00	−1.00−0.92	Rank-biserial r	Large
Platelet	Normal vs. IDA+CD	−1.02	−1.62−0.41	Cohen's d	Large
RDW-CV	Normal vs. IDA+CD	0.90	0.56–1.00	Rank-biserial r	Large

Effect sizes were calculated as Cohen's d for parametric comparisons or Rank-biserial correlation (r) for non-parametric comparisons. Magnitude interpretation: |d| or |r| ~0.2 = Small, ~0.5 = Medium, ~0.8 = Large.

### 3.3 Comparative analysis and correlations

**Figure 2:**
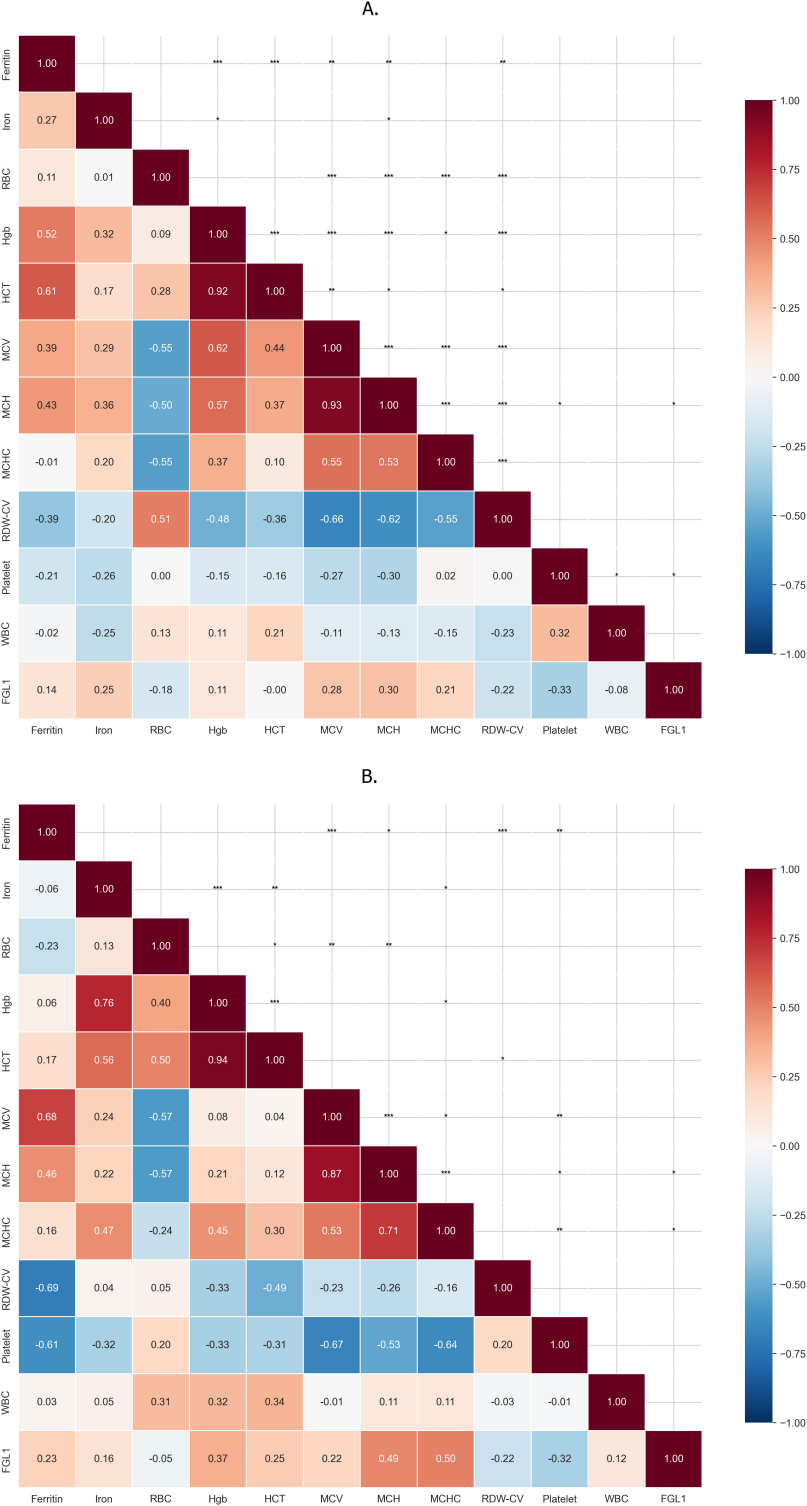
Correlation patterns of FGL1 with hematological and iron parameters. Correlation matrices illustrating relationships among hematological indices, iron parameters, and serum FGL1 levels in **(A)** patients with primary iron deficiency anemia (IDA) and **(B)** patients with iron deficiency anemia associated with chronic disease (IDA+CD). Color intensity reflects the strength and direction of correlation (Spearman's rho), with red indicating positive and blue indicating negative associations. Statistically significant correlations are denoted by asterisks (*p* < 0.05, *p* < 0.01, *p* < 0.001). Distinct correlation patterns highlight differential biomarker interactions in isolated IDA vs. IDA in the context of chronic disease.

Following the comparison of baseline parameters, correlation matrices were constructed to further characterize the relationships between hematological parameters, iron status and FGL1 levels. In the IDA group (Figure 2A), classical iron markers showed the expected associations, with ferritin and serum iron demonstrating moderate correlations with hemoglobin (*r* = 0.52 and *r* = 0.32, respectively). Strong internal clustering was observed among red cell indices, particularly between MCV and MCH (*r* = 0.93). RDW showed marked negative correlations with MCV (*r* = −0.66) and MCH (*r* = −0.62), confirming heterogeneous microcytosis. Within this context, FGL1 showed only weak associations with hematological and iron parameters, including mild correlations with serum iron (*r* = 0.25) and MCH (*r* = 0.30).

In the IDA+CD group (Figure 2B) serum iron retained a strong correlation with hemoglobin (*r* = 0.76), whereas ferritin showed minimal association (*r* = 0.06). Platelets showed a strong negative correlation with MCV (*r* = −0.67). Fibrinogen-like protein 1 demonstrated moderate correlations with hemoglobinization indices such as MCH (*r* = 0.49). Correlation coefficients with corresponding 95% confidence intervals are provided in Supplementary Table S1.

### 3.4 Diagnostic performance of FGL1

**Figure 3:**
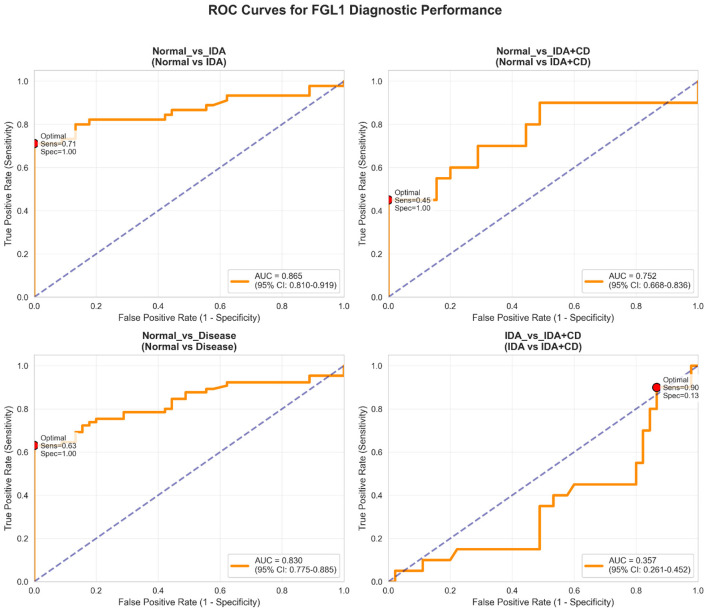
Receiver operating characteristic (ROC) curves evaluating the diagnostic performance of serum FGL1 for distinguishing: (upper left) healthy controls from primary IDA, (upper right) healthy controls from IDA+CD, (lower left) healthy controls from all anemic patients combined, and (lower right) primary IDA from IDA+CD. Areas under the curve (AUC) with 95% confidence intervals are shown for each comparison. Optimal cut-off points (red dots) indicate thresholds maximizing specificity and sensitivity.

ROC analysis demonstrated strong discrimination between healthy controls from primary IDA (*AUC* = 0.865; 95% CL: 0.819–0.919) and between controls and the combined disease cohort (*AUC* = 0.830; 95% CI: 0.775–0.885) (Figure 3). Diagnostic performance was moderate for distinguishing controls from IDA+CD (*AUC* = 0.752; 95% CI: 0.668–0.836). Fibrinogen-like protein 1 showed poor discrimination between primary IDA and IDA+CD (*AUC* = 0.357; 95% CI: 0.261–0.452). Optimal thresholds yielded high specificity (100%) but variable sensitivity across comparisons.

### 3.5 Multivariate biomarker profiling via principal component analysis

**Figure 4:**
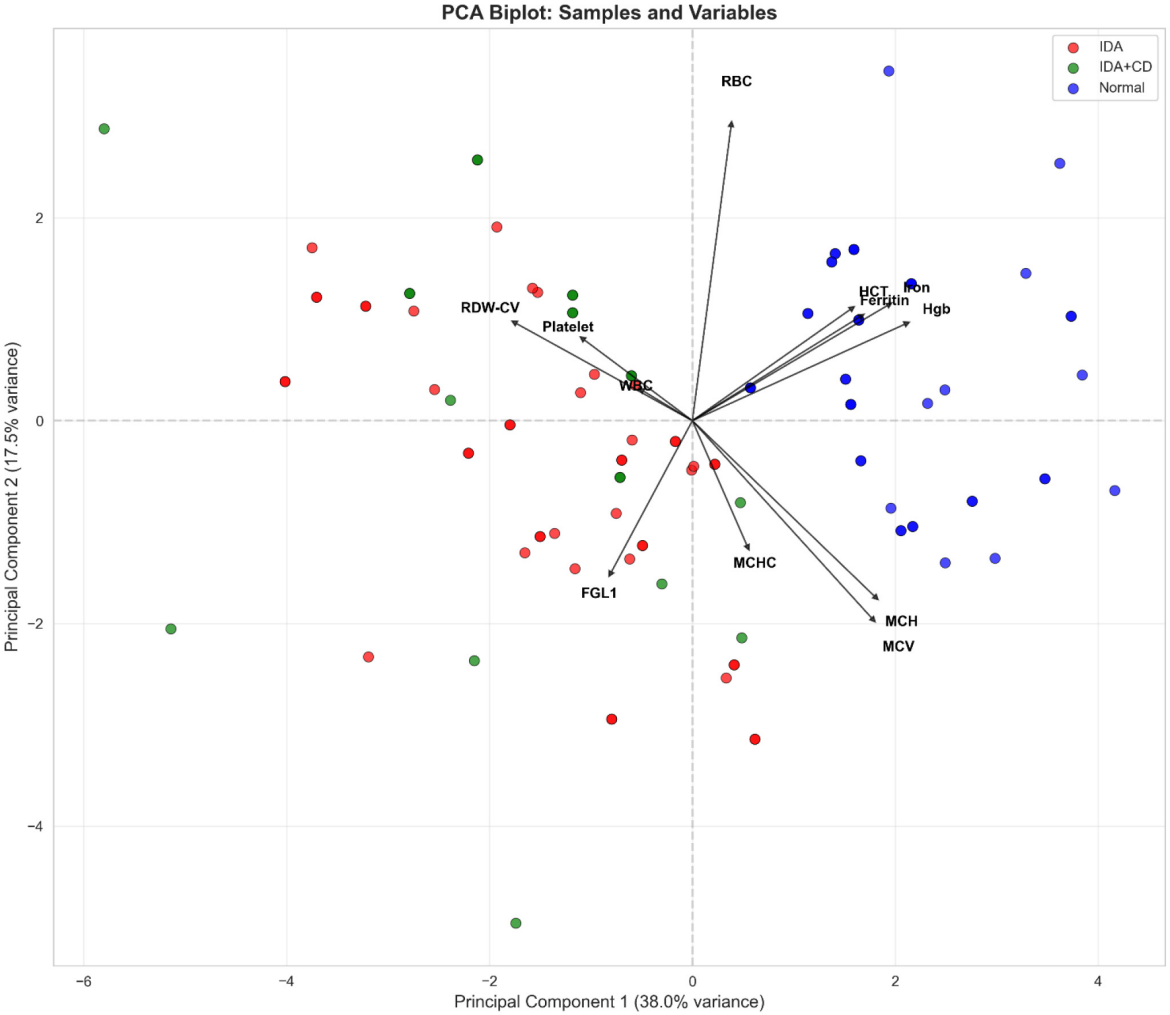
Principal component analysis of hematological and iron-related biomarkers.

To examine the interrelated structure of hematological and iron-related biomarkers, PCA was performed. The biplot of the first two principal components (PC), which together explained 55.5% of the total variance (PC1: 38.0%, PC2: 17.5%), is presented in Figure 4.

PC1 captured the iron-deficiency signature, with strong loadings from hemoglobin, ferritin, red cell indices, and FGL1, and clearly separated healthy controls from both IDA groups. Principal component 2, driven primarily by WBC and platelet counts, introduced overlap between IDA and IDA+CD. Fibrinogen-like protein 1 clustered with markers of iron-restricted erythropoiesis along PC1, indicating its alignment with the broader biochemical pattern of ID.

## 4 Discussion

This study provides the first clinical evidence that circulating FGL1 levels are significantly higher in patients with IDA compared with healthy individuals. The increased levels of FGL1 were observed against the expected physiological context of hypochromic, microcytic anemia with markedly depleted iron stores. Although FGL1 levels were higher in primary IDA than in IDA+CD, this difference did not reach statistical significance. These findings indicate that FGL1 levels reflects iron-restricted erythropoiesis but is also influenced by the presence or absence of chronic disease.

The increased FGL1 in IDA is consistent with recently described preclinical mechanism. Experimental data identify FGL1 as a hypoxia-inducible hepatokine that acts as a soluble antagonist of the BMP signaling pathway in the liver (21). Iron regulation depends on the BMP6–SMAD–hepcidin axis, in which liver sinusoidal endothelial cells release BMP6 in response to hepatocellular iron status, thereby activating hepcidin transcription in neighboring hepatocytes (23). Experimental studies suggest that FGL1 can bind and neutralize BMP6, reducing hepcidin expression and thereby promoting iron mobilization for erythropoiesis (21). Although hepcidin was not measured in the present study, the observed increase in FGL1 in ID is consistent with this proposed regulatory pathway.

Our results differ from a recent study in patients with end-stage chronic kidney disease on hemodialysis, which reported reduced FGL1 levels compared with healthy controls (22). In that context, renal failure, uremic toxin accumulation, chronic inflammation, and hepatic dysfunction may profoundly alter hepatokine production and clearance. In contrast, renal function was largely preserved in our cohort, including within IDA+CD, where only a minority of patients had CKD, and none required dialysis. These differences suggest that FGL1 regulation depends not only on iron status and hypoxia but also on organ-specific disease, particularly hepatic and renal integrity.

The present findings help link mechanistic hypotheses to clinical physiology. In primary IDA, hepcidin suppression is an appropriate adaptive response that enables iron mobilization from stores and enhances intestinal absorption (24). The concomitant increase in FGL1 suggests the presence of an additional hepatokine-based regulatory layer that supports this process. Fibrinogen-like protein 1 may therefore function as a hepatocyte-derived counterpart to ERFE, with a shared physiological goal of improving iron availability but distinct molecular regulation.

### 4.1 Correlation analysis

A key observation was the dissociation between elevated FGL1 levels and classical systemic iron markers. In primary IDA, FGL1 did not correlate meaningfully with serum iron or ferritin, indicating that it does not directly reflect iron stores. Instead, modest positive correlations with MCV and MCH suggest that FGL1 may more closely track hemoglobinization within erythroid precursors or erythropoietic demand than circulating iron indices.

In IDA+CD, FGL1 demonstrated stronger associations with hemoglobinization parameters, particularly MCH (*r* = 0.49) and MCHC (*r* = 0.50), despite the potential influence of chronic disease. Ferritin showed minimal correlation with hemoglobin, whereas serum iron retained a strong association. Although patients with markedly elevated ferritin or increased CRP were excluded to avoid confounding by acute inflammation, residual low-grade inflammatory effects inherent to chronic disease may still alter ferritin behavior and iron mobilization. These patterns suggest that FGL1 continues to reflect erythropoietic activity in the presence of chronic disease, but its absolute concentration may be modulated by subtle inflammatory signaling even within a clinically screened cohort.

One plausible explanation for the lower FGL1 levels in IDA+CD relative to primary IDA, despite similar ID, is the interplay between hypoxia-driven and inflammation-driven hepatic pathways. Hypoxia and ID induce FGL1 through HIF-dependent mechanisms, whereas inflammatory cytokines such as IL-6 can also affect hepatokine synthesis (25). Chronic inflammation, however, creates a hepatic environment dominated by IL-6–STAT3 activation and hepcidin induction, which opposes the hepatocellular signals that favor hepcidin suppression (26). This competing network may limit FGL1 induction in IDA+CD leading to intermediate levels and reducing separation from primary IDA. The IDA+CD group was older and clinically heterogeneous, with multiple overlapping comorbid conditions. Given the modest subgroup size and the absence of detailed organ-specific functional data, the observed FGL1 pattern in this cohort may reflect the combined influence of iron deficiency, age, and overall comorbidity burden rather than a disease-specific regulatory effect. Accordingly, interpretations regarding chronic disease-related modulation of FGL1 should be made with caution.

### 4.2 Diagnostic performance

ROC analysis demonstrates that FGL1 achieves excellent specificity, reaching 100% at optimal thresholds across all normal-vs.-disease comparisons. The high specificity observed at the optimal threshold suggests potential utility of FGL1 in identifying iron-deficient state; however, its diagnostic role requires validation in larger prospective studies. The AUC of 0.865 indicates good discriminatory performance for distinguishing healthy individuals from patients with primary IDA. However, the reduced AUC for normal vs. IDA + CD (0.752) and the poor discrimination between IDA and IDA + CD (0.357) highlight an important diagnostic constraint. Direct comparison with established biomarkers such as ferritin was not performed; therefore, the diagnostic performance of FGL1 should be interpreted as exploratory.

### 4.3 Multivariate structure

Principal component analysis provides additional insight into this behavior. Fibrinogen-like protein 1 aligns closely with biomarkers of iron-restricted erythropoiesis along PC1, which separates healthy controls from both IDA groups and represents the dominant iron-deficiency axis. PC2, influenced primarily by WBC and platelet counts, introduces overlap between IDA and IDA+CD. This pattern mirrors the ROC findings and supports the interpretation that FGL1 is best understood as part of an iron-deficiency signature rather than as a marker capable of discriminating anemia subtypes on its own. PCA is an exploratory, variance-based technique, and the observed alignment of FGL1 with erythropoietic and iron-related variables reflects shared statistical variance rather than a direct mechanistic relationship. These findings should therefore be interpreted as hypothesis-generating rather than evidence of causal pathway interactions.

In clinical settings where inflammation compromises the interpretability of ferritin and other traditional biomarkers, FGL1 may thus serve as a useful component of multivariate diagnostic models. Interpreting FGL1 alongside inflammatory markers such as CRP, hepcidin, or IL-6 may improve etiological classification in patients with complex anemia.

### 4.4 Clinical implications

The present study suggests several clinical implications. First, FGL1 demonstrates robust discriminatory capacity for identifying iron-deficient states and retains diagnostic value when ferritin is potentially confounded by inflammation. This profile supports its role as a complementary biomarker in the initial evaluation of anemia, particularly in primary care or resource-limited settings where extensive inflammatory profiling may not be available. Second, the limited ability of FGL1 to distinguish primary IDA from IDA+CD indicates that it should not be used as a standalone tool for etiological classification. Instead, FGL1 may be most informative within a panel that integrates iron indices and inflammatory markers, guiding more precise diagnosis and treatment decisions.

## 5 Strengths, limitations, and future directions

This study offers several strengths. It includes clearly defined patient groups with rigorously verified diagnostic criteria and applies comprehensive statistical methods to characterize the clinical and biochemical profiles of ID. The study introduces FGL1 as a novel hepatokine within human iron biology and provides early clinical evidence supporting its association with iron-restricted erythropoiesis. The multivariate analytical approach strengthens the interpretation of FGL1 as part of an integrated biomarker signature rather than an isolated analyte.

Despite these strengths, several limitations must be acknowledged. The cross-sectional design does not allow causal inference and cannot determine whether elevated FGL1 represents a primary regulatory signal, a secondary adaptive response, or an epiphenomenon linked to metabolic or inflammatory stress.

Although the overall sample size supports group comparisons, the smaller number of patients with IDA in the context of chronic disease limited the statistical power to detect moderate differences between primary IDA and IDA+CD and reduced the precision of subgroup estimates. Correlation estimates and diagnostic performance measures within the IDA+CD group should therefore be interpreted cautiously.

In addition, demographic heterogeneity between study groups represents a potential source of confounding. Patients with primary IDA were younger than those with IDA+CD, and a marked female predominance was observed across the anemia cohorts. Because age and sex may influence hepatokine expression and iron-related parameters, differences in FGL1 concentrations may partly reflect demographic variation in addition to disease-related effects. The sample size and subgroup structure did not permit reliable multivariable adjustment; therefore, this potential influence should be considered when interpreting group comparisons.

The absence of cytokine profiling and direct hepcidin measurements restricts mechanistic interpretation. Consequently, the proposed interaction between FGL1 and the hepcidin regulatory network, including the BMP-SMAD pathway, remains inferential and is supported by prior experimental evidence rather than direct demonstration in this cohort. This constraint also limits the evaluation of competing inflammation-mediated and hypoxia-driven regulatory pathways. A subset of participants had type 2 diabetes mellitus, a condition known to modulate hepatokine secretion; although the data did not indicate a measurable influence on FGL1, the study was not powered to evaluate metabolic effects. The possibility of diabetes-related modulation therefore remains open.

This was a single-center study, and participants were identified from a hospital-based population, which may introduce selection bias and limit representativeness of the broader community. Patients with IDA were identified through a clinical laboratory database and therefore may reflect a referral-based disease spectrum. In addition, healthy controls were recruited from hospital staff and community volunteers, which may introduce a healthy-worker effect and reduce the likelihood of underlying comorbidities. Furthermore, the study population was drawn from a single geographic region, and ethnic or population-specific differences in hepatokine regulation cannot be excluded. These factors may limit the generalizability of the findings to other populations and clinical settings.

Future research should investigate the longitudinal behavior of FGL1 during iron supplementation, erythropoietic recovery, and anti-inflammatory therapy to determine whether it provides value as a dynamic biomarker of treatment response. Studies that examine the interaction between FGL1 and key regulatory pathways, including hepcidin, ferroportin, IL-6, and hypoxia-inducible signaling may clarify its mechanistic role within iron homeostasis. Incorporating inflammatory indices and hepcidin measurements into multivariate diagnostic models may improve etiological classification of anemia in complex clinical presentations. Larger, multi-center studies are required to validate the generalizability and clinical utility of FGL1 across diverse demographic and pathological contexts.

## 6 Conclusion

This study identifies FGL1 as a hepatokine that shows substantial increase in patients with IDA and demonstrates distinct concentration patterns across etiological categories. Its strong diagnostic performance, particularly its high specificity, and its integration into the multivariate structure of iron-deficient states support a biological association of FGL1 with the adaptive response to iron depletion. The findings indicate that FGL1 does not behave as a nonspecific acute-phase reactant and are consistent with a potential role in pathways that influence hepcidin and iron mobilization. However, direct interaction with hepcidin or inflammatory mediators was not assessed in this study. Further mechanistic and longitudinal studies are required to clarify its regulatory role and to determine its clinical utility as a diagnostic or monitoring biomarker in anemia.

## Data Availability

The original contributions presented in the study are included in the article/Supplementary material, further inquiries can be directed to the corresponding author.
