# Supplementary material for: Evaluation of FGL1 as a hepatokine marker in iron deficiency
Source: Front Nutr. 2026 Mar 6;13:1767385. doi: 10.3389/fnut.2026.1767385 (PMC13002365; doi:10.3389/fnut.2026.1767385)
Supplement: Supplementary file 1 [file Table_1.docx]

Supplementary Material

Supplementary Table S1. Correlation coefficients between FGL1 and selected hematological and iron parameters

A. Primary IDA group (n = 46)

| Variable | Correlation (r) | 95% CI | p-value |
| --- | --- | --- | --- |
| Hemoglobin | 0.11 | −0.19 to 0.39 | 0.46 |
| Serum iron | 0.24 | −0.04 to 0.50 | 0.100 |
| Ferritin | 0.14 | −0.16 to 0.42 | 0.34 |
| MCV | 0.27 | −0.02 to 0.52 | 0.06 |
| MCH | 0.29 | 0.003 to 0.54 | 0.045 |
| MCHC | 0.21 | −0.09 to 0.47 | 0.16 |
| RDW-CV | −0.22 | −0.47 to 0.08 | 0.14 |
| Platelet count | −0.33 | −0.56 to −0.05 | 0.02 |
| WBC count | −0.08 | −0.36 to 0.23 | 0.59 |

B. IDA with chronic disease (n = 20)

| Variable | Correlation (r) | 95% CI | p-value |
| --- | --- | --- | --- |
| Hemoglobin | 0.37 | −0.09 to 0.70 | 0.11 |
| Serum iron | 0.16 | −0.31 to 0.56 | 0.49 |
| Ferritin* | 0.23 | −0.23 to 0.62 | 0.33 |
| MCV | 0.22 | −0.25 to 0.60 | 0.34 |
| MCH | 0.49 | 0.06 to 0.77 | 0.03 |
| MCHC | 0.50 | 0.08 to 0.77 | 0.03 |
| RDW-CV | −0.22 | −0.61 to 0.24 | 0.34 |
| Platelet count | −0.32 | −0.67 to 0.16 | 0.16 |
| WBC count | 0.12 | −0.34 to 0.53 | 0.61 |
